# FDTool: a Python application to mine for functional dependencies and candidate keys in tabular data

**DOI:** 10.12688/f1000research.16483.2

**Published:** 2019-06-19

**Authors:** Matt Buranosky, Elmar Stellnberger, Emily Pfaff, David Diaz-Sanchez, Cavin Ward-Caviness

**Affiliations:** 1 National Health and Environmental Effects Research Laboratory, United States Environmental Protection Agency, Chapel Hill, NC, USA; 2 University of Klagenfurt, Klagenfurt, Austria; 3 University of North Carolina at Chapel Hill, Chapel Hill, NC, USA

**Keywords:** Functional dependencies, Data mining, Electronic health records, Relational database, FDTool, Rule discovery

## Abstract

Functional dependencies (FDs) and candidate keys are essential for table decomposition, database normalization, and data cleansing. In this paper, we present FDTool, a command line Python application to discover minimal FDs in tabular datasets and infer equivalent attribute sets and candidate keys from them. The runtime and memory costs associated with seven published FD discovery algorithms are given with an overview of their theoretical foundations. Previous research establishes that FD_Mine is the most efficient FD discovery algorithm when applied to datasets with many rows (> 100,000 rows) and few columns (< 14 columns). This puts it in a special position to rule mine clinical and demographic datasets, which often consist of long and narrow sets of participant records. The structure of FD_Mine is described and supplemented with a formal proof of the equivalence pruning method used. FDTool is a re-implementation of FD_Mine with additional features added to improve performance and automate typical processes in database architecture. The experimental results of applying FDTool to 13 datasets of different dimensions are summarized in terms of the number of FDs checked, the number of FDs found, and the time it takes for the code to terminate. We find that the number of attributes in a dataset has a much greater effect on the runtime and memory costs of FDTool than does row count. The last section explains in detail how the FDTool application can be accessed, executed, and further developed.

## Introduction

Functional dependencies (FDs) are key to understanding how attributes in a database schema relate to one another. An FD defines a rule constraint between two sets of attributes in a relation ^1^ *r*( *U*), where *U* = { *v*_1_, *v*_2_,…, *v*_*m*_} is a finite set of attributes ( Yao *et al.*, 2002). A combination of attributes over a dataset is called a *candidate* ( Yao *et al.*, 2002). An FD *X* → *Y* asserts that the values of candidate *X* uniquely determine those of candidate *Y* ( Yao *et al.*, 2002). For example, the social security number (SSN) attribute in a dataset of public records functionally determines the first name attribute. Because the FD holds, we write { *SSN*} → { *first*_ *name*}.

**Definition 1.** A *functional dependency X* → *Y*, where *X*, *Y* ⊆ *U*, is satisfied by *r*( *U*), if for all pairs of tuples *t _i_*, *t _j_*∈ *r*( *U*), we have that *t _i_* [ *X*] = *t _j_* [ *X*] implies *t _i_* [ *Y*] = *t _j_* [ *Y*] ( Yao & Hamilton, 2008).

In this case, *X* is the *left-hand side* of an FD, and *Y* is the *right-hand side* ( Yao *et al.*, 2002). If *Y* is not functionally dependent on any proper subset of *X*, then *X* → *Y* is *minimal* ( Yao *et al.*, 2002). Minimal FDs are our only concern in rule mining FDs, since all other FDs are logically implied. For instance, if we know { *SSN*} → { *first*_ *name*}, then we can infer that { *SSN*, *last*_ *name*} → { *first*_ *name*}.

### Power set lattice

**Figure 1.**
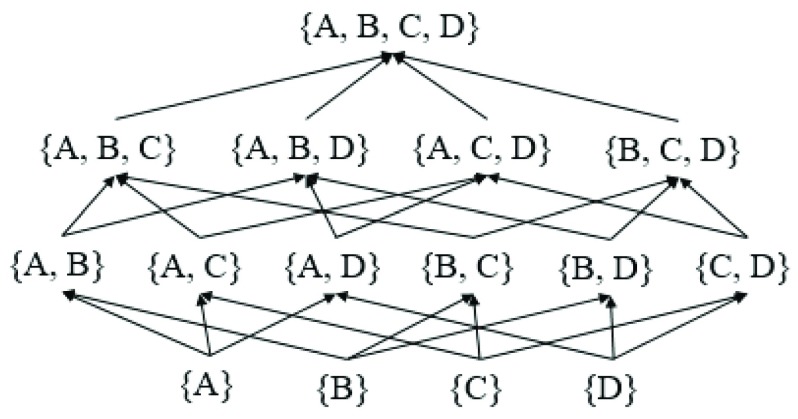
Nonempty combinations of attributes *A*, *B*, *C*, and *D* by k-level.

The search space for FDs can be represented as a *power set lattice* of nonempty attribute combinations. Figure 1 gives the nonempty attribute combinations of a relation *r*( *U*) such that *U* = { *A*, *B*, *C*, *D*}. There are 2 ^*n*^ – 1 = 2 ^4^ – 1 = 15 attribute subsets in the power set lattice ( Yao & Hamilton, 2008). Each combination *X* of the attributes in *U* can be the left-hand side of an FD *X* → *Y* such that *X* → *Y* is satisfied by relation *r*( *U*) ( Yao & Hamilton, 2008). Since the attribute set itself *U* trivially determines each one of its proper subsets, it can be ignored as a candidate. There remain 2 ^*n*^ – 2 = 2 ^4^ – 2 = 14 nonempty subsets of *U* that are to be considered candidates.

There are *n* · 2 ^*n*–1^ – *n* = 4 · 2 ^4–1^ – 4 = 28 edges (or arrows) in the semi-lattice of the complete search space for FDs in relation *r*( *U*) ( Yao & Hamilton, 2008). The size of the search space for FDs is exponentially related to the number of attributes in *U*. Hence, the search space for FDs increases quite significantly when there is a greater number of attributes in *U*. For instance, when there are 12 attributes in a relation, the search space for FDs climbs to 24,564. This gives reason to be cautious of runtime and memory costs when deploying a rule mining algorithm to discover FDs.

### Partition

The algorithms used to discover FDs differ in their approach to navigating the complete search space of a relation. Their candidate pruning methods vary and sometimes the methods used to validate FDs do as well. These differences affect runtime and memory behavior when used to process tables of different dimensions.

A common data structure used to validate FDs is the partition. A partition places tuples that have the same values on an attribute into the same group ( Yao *et al.*, 2002).

**Definition 2.** Let *X* ⊆ *U* and let *t*_1_,…, *t _n_* be all the tuples in a relation *r*( *U*). The *partition* over *X*, denoted ∏ _*X*_, is a set of the groups such that *t _i_* and *t _j_*, 1 ≤ *i*, *j* ≤ *n*, *i*≠ *j*, are in the same group if and only if *t _i_* [ *X*] = *t _j_* [ *X*] ( Yao *et al.*, 2002).

It follows from Definition 2 that the *cardinality of the partition* **card**(∏ _*A*_( *r*)) is the number of groups in partition ∏ _*A*_ ( Yao & Hamilton, 2008). The cardinality of the partition offers a quick approach to validating FDs in a dataset.

**Theorem 1.** An FD *X* → *Y* is satisfied by a relation *r*( *U*) if and only if **card**(∏ _*X*_) = **card**(∏ _*XY*_) ( Huhtala *et al.*, 1999).

Theorem 1 provides an efficient method to check whether an FD *X* → *Y* holds in a relation ^2^. Huhtala *et al.* (1999) proved it to support a fast validation method for relations consisting of a large number of tuples.

### Closure

Efforts in relational database theory have led to more runtime and memory efficient methods to check the complete search space of a relation for FDs. In place of needing each arrow in a semi-lattice checked, we can infer the FDs that logically follow from those already discovered. Such FDs are to be discovered as a consequence of *Armstrong’s Axioms* ( Maier, 1983) and the inference axioms derivable from them ( Ramakrishnan & Gehrke, 2000), which are

– *Reflexivity*: *Y* ⊆ *X* implies *X* → *Y*;
– *Augmentation*: *X* → *Y* implies *XZ* → *YZ*;
– *Transitivity*: *X* → *Y* and *Y* → *Z* imply *X* → *Z*;
– *Union*: *X* → *Y* and *X* → *Z* imply *X* → *YZ*;
– *Decomposition*: *X* → *YZ* implies that *X* → *Y* and *X* → *Z*.

These axioms signal the distinction between FDs that can be inferred from already discovered FDs and those that cannot ( Maier, 1983). Exploiting what can be derived from Armstrong’s Axioms allows us to avoid having to check many of the candidates in a search space.

**Definition 3.** Let *F* be a set of functional dependencies over a dataset *D* and *X* be a candidate over *D*. The *closure of candidate X* with respect to *F*, denoted *X* ^+^, is defined as { *Y* | *X* → *Y* can be deduced from *F* by Armstrong’s Axioms} ( Yao & Hamilton, 2008).

The *nontrivial closure* ^3^ *of candidate X* with respect to *F* is defined as X* = *X* ^+^ \ *X* and written X* ( Yao & Hamilton, 2008). Definition 3 gives room to elegantly define keys. Informally, a key implies that a relation does not have two distinct tuples with the same values on those attributes. Keys uniquely identify all tuple records in a dataset.

**Definition 4.** Let *R* be a relational schema and *X* be a candidate of *R* over a dataset *D*. If *X* ∪ X* = *R*, then *X* is a *key* ( Yao *et al.*, 2002).

A *candidate key X* of a relation is a minimal key for that relation. This means that there is no proper subset of *X* for which Definition 4 holds.

## Rule mining algorithms

Existing functional dependency algorithms are split between three categories: Difference- and agree-set algorithms (e.g., Dep-Miner, FastFDs), Dependency induction algorithms (e.g., FDEP), and Lattice traversal algorithms (e.g., TANE, FUN, FD_Mine, DFD) ( Papenbrock *et al.*, 2015).

*Difference- and agree-set algorithms* model the search space of a relation as the cross product of all tuple records ( Papenbrock *et al.*, 2015). They search for sets of attributes agreeing on the values of certain tuple pairs. Attribute sets only functionally determine other attribute sets whose tuple pairs agree, i.e., *agree-sets* ( Asghar & Ghenai, 2015; Papenbrock *et al*., 2015). Then, agree-sets are used to derive all minimal FDs.

*Dependency induction algorithms* assume a base set of FDs in which each attribute functionally determines each other attribute ( Papenbrock *et al.*, 2015). While iterating through row data, observations are made that require certain FDs to be removed from the base set and others added to it. These observations are made by comparing tuple pairs based on the equality of their projections. After each record in a dataset is compared, the FDs left in the base set are considered valid, minimal and complete ( Papenbrock *et al.*, 2015).

*Lattice traversal algorithms* model the search space of a relation as a power set lattice. Most of such algorithms, (i.e., TANE, FUN, FD_Mine) use a level-wise approach to traversing the search space of a relation from the bottom-up ( Papenbrock *et al.*, 2015). They start by checking ^4^ for FDs that are singleton sets on the left-hand side and iteratively transition to candidates of greater cardinality.

### Performance

Papenbrock *et al.* (2015) released an experimental comparison of the aforementioned FD discovery algorithms. The seven algorithms were re-implemented in Java based on their original publications and applied to 17 datasets of various dimensions. They found that none of the algorithms are suited to yield the complete result set of FDs from a dataset consisting of 100 columns and 1 million rows ( Papenbrock *et al.*, 2015). Hence, it is a matter of discretion to choose the algorithm best fitting the dimensions of a dataset.

The experimental results show that lattice traversal algorithms are the least memory efficient, since each *k*-level ^5^ can be a factor greater than the size of the previous level ( Papenbrock *et al.*, 2015). Difference- and agree-set algorithms and dependency induction algorithms perform favorably in memory experiments as a result of their operating directly on data and efficiently storing result sets. Lattice traversal algorithms scale poorly on tables with many columns (≥ 14 columns) due to memory limits ( Papenbrock *et al.*, 2015).

Lattice traversal algorithms are the most effective on datasets with many rows, because their validation method ^6^ operates on attribute sets as opposed to data ( Papenbrock *et al.*, 2015). This puts such algorithms in a special position to rule mine clinical and demographic record datasets, which often consist of long and narrow sets of participant records. Difference- and agree-set algorithms and dependency induction algorithms commonly reach time limits when applied to datasets of these dimensions (> 100,000 rows) ( Papenbrock *et al.*, 2015).

### Lattice traversal algorithms

Lattice traversal algorithms iterate through *k*-levels represented in a power set lattice. If the lattice is traversed from the bottom-up, we say the algorithm is *level-wise*.

**Definition 5.** Let *X*_1_, *X*_2_,…, *X _k_*, *X _k_*_+1_ be ( *k* + 1) attributes over a database *D*. If *X*_1_ *X*_2_… *X _k_* → *X _k_*_+1_ is an FD with *k* attributes on its left hand side, then it is called a *k-level* FD ( Yao *et al.*, 2002).

The search space for FDs is reduced at the end of each iteration using pruning rules. *Pruning rules* check the validity of candidates not yet checked with FDs already discovered and those inferred from Armstrong’s Axioms ( Yao & Hamilton, 2008). After a search space is pruned, an *Apriori_Gen* principle generates *k*-level candidates with the ( *k* – 1)-level candidates that were not pruned ( Yao & Hamilton, 2008).

**Apriori_Gen**:

– *oneUp*: generates all possible candidates in *C _k_* from those in *C _k_*_–1_.
– *oneDown*: generates all possible candidates in *C _k_*_–1_ from those in *C _k_*.

Level-wise lattice traversal algorithms stop iterating after all candidates in a search space are pruned. In this case, *Apriori_Gen* generates the null set ∅ raising a flag for the algorithm to terminate. This has the effect of shortening runtime to the degree that FDs are discovered and others are inferred.

### Tane

The level-wise lattice traversal algorithms TANE, FUN, and FD_Mine differ in terms of pruning rules. FUN and FD_Mine expand on the pruning rules of TANE. Released by Huhtala *et al.* (1999), TANE prunes a search space on the basis that only minimal and non-trivial ^7^ FDs need be checked. TANE restricts the right-hand side candidates *C* ^+^ for each attribute combination *X* to the set

$$C^{+}(X)=\left\{A\in R|\forall B\in X\;:\;X\;\backslash \;\left\{A,B\right\}\to B\;\text{does}\;\text{not}\;\text{hold}\right\},$$

which contains all the attributes that the set *X* may still functionally determine ( Papenbrock *et al.*, 2015). The set *C* ^+^ is used in the following pruning rules ( Papenbrock *et al.*, 2015).

• **Minimality pruning:** If an FD *X* \ *A* → *A* holds, *A* and all *B* ∈ *C* ^+^ ( *X*) \ *X* can be removed from *C* ^+^ ( *X*).

• **Right-hand side pruning:** If *C* ^+^ ( *X*) = ∅, the attribute combination *X* can be pruned from the lattice, as there are no more right-hand side candidates for a minimal FD.

• **Key pruning:** If the attribute combination *X* is a key, it can be pruned from the lattice.

Key pruning implies that all supersets of a key, i.e., *super keys*, can be removed, since they are by definition non-minimal ( Huhtala *et al.*, 1999).

## FD_Mine

Like TANE and FUN, FD_Mine is structured around the level-wise lattice traversal approach and the aforementioned pruning rules. Unlike the other two algorithms, FD_Mine, authored by Yao *et al.* (2002), uses the concept of equivalence as means to more exhaustively prune the search space of a candidate ( Papenbrock *et al.*, 2015). Informally, attribute sets are equivalent if and only if they are functionally dependent on each other ( Papenbrock *et al.*, 2015).

The proofs demonstrating that no useful information is lost in pruning candidates from equivalent attribute sets are reproduced in this section and were originally developed by Yao & Hamilton (2008). The equivalence pruning method can be derived directly from Armstrong’s Axioms.

**Definition 6.** Let *X* and *Y* be candidates over a dataset *D*. If *X* → *Y* and *Y* → *X* hold, then we say that *X* and *Y* are an *equivalence* and denote it as *X* ↔ *Y*.

After a *k*-level is fully validated, i.e., each *k*-level candidate is checked, FD_Mine determines equivalent attribute sets using the FDs already discovered.

**Theorem 2.** Let *X*, *Y* ⊆ *U*. If *Y* ⊆ *X* ^+^ and *X* ⊆ *Y* ^+^, then *X* ↔ *Y* ( Yao & Hamilton, 2008).

*Proof.* Since *X* → *X* ^+^ and *Y* ⊆ *X* ^+^, Decomposition implies that *X* → *Y*. By a similar argument, *Y* → *X* holds. Because *X* → *Y* and *Y* → *X*, we have by definition that *X* ↔ *Y* holds.

Lemma 3 and Lemma 4 are derived from Armstrong’s Axioms with the assumption of the equivalence *X* ↔ *Y*.

**Lemma 3.** Let *W*, *X*, *Y*, *Y*', *Z* ⊆ *U* and *Y* ⊆ *Y*'. If *X* ↔ *Y* and *XW* → *Z*, then *Y*' *W* → *Z* ( Yao & Hamilton, 2008).

*Proof.* Suppose that *X* ↔ *Y* and *XW* → *Z*. This implies that *X* → *Y*. By Augmentation, *YW* → *XW*. By Transitivity, *YW* → *XW* and *XW* → *Z* give that *YW* → *Z*. By Augmentation, *Y*' \ *Y* can be added to both sides of *YW* → *Z* to give that *YW*( *Y*' \ *Y*) → *Z*( *Y*' \ *Y*). By *Y* ⊂ *Y*', we know that *Y*' *W* → *Z*( *Y*' \ *Y*). Then, by Decomposition, *Y*' *W* → *Z*.

**Lemma 4.** Let *W*, *X*, *Y*, *Z* ⊆ *U*. If *X* ↔ *Y* and *WZ* → *X*, then *WZ* → *Y* ( Yao & Hamilton, 2008).

*Proof.* By *X* ↔ *Y*, we know that *X* → *Y*. By Transitivity, *WZ* → *X* and *X* → *Y* imply *WZ* → *Y*.

Theorem 2 checks attribute sets *X* and *Y* for the equivalence *X* ↔ *Y*. FD_Mine assumes that the attribute set *Y* is generated before *X*. By Lemma 3 and Lemma 4, we know that for equivalence *X* ↔ *Y*, no further attribute sets *Z* such that *Y* ⊆ *Z* need be checked ( Yao & Hamilton, 2008). Hence, *Y* is deleted as a result of the following pruning rule.

• **Equivalence pruning:** If *X* ↔ *Y* is satisfied by relation *r*( *U*), then candidate *Y* can be deleted. ( Yao & Hamilton, 2008).

Exploiting the equivalence pruning method leaves FD_Mine in a more aggressive position to prune candidates than TANE. This offers an advantage in terms of runtime and memory behavior ( Yao *et al*., 2002).

### Non-minimal FDs

**Figure 2.**
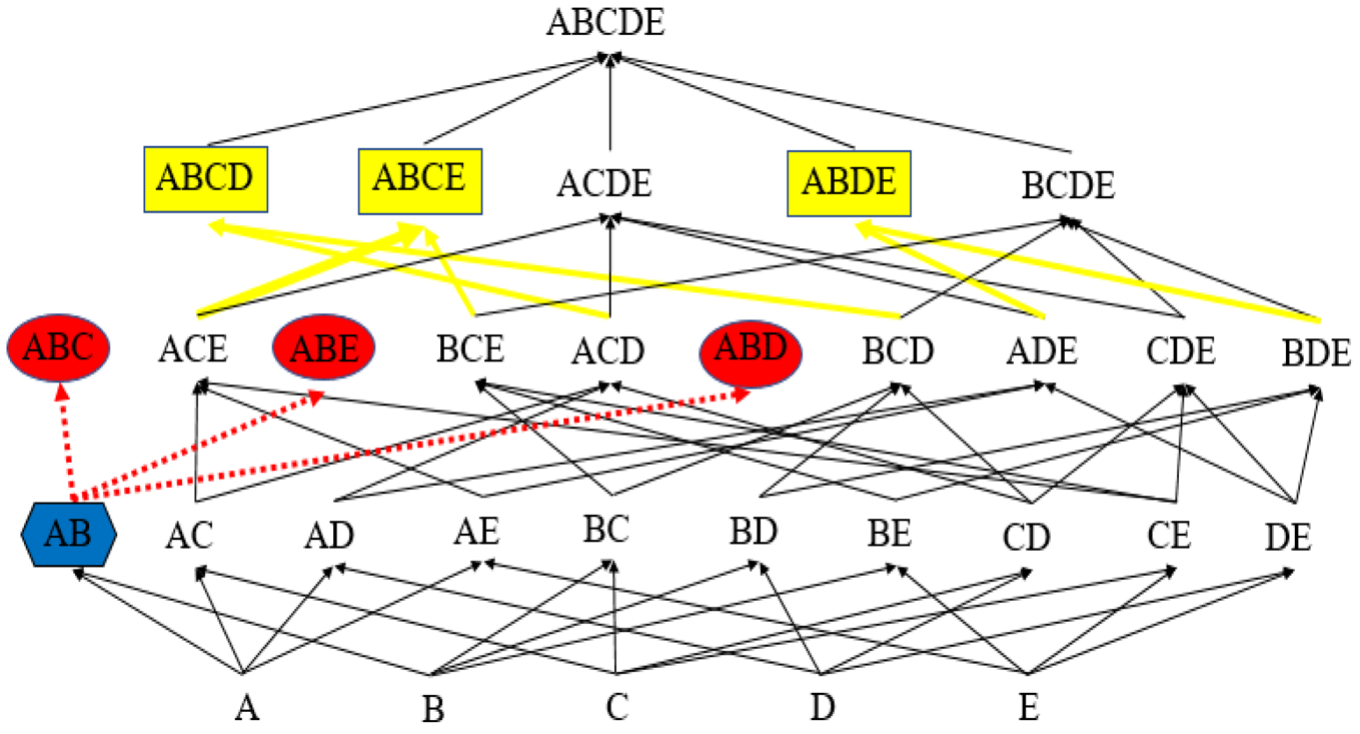
A pruned power set lattice. FD_Mine deletes the candidates *ABC*, *ABE*, and *ABD* (red ovals) as a result of finding the candidate key *AB* (blue hexagon). It generates supersets of *AB* (yellow rectangles) at the next level.

The pseudo-code proposed in the second version of FD_Mine ( Yao & Hamilton, 2008) will under certain circumstances output non-minimal FDs ( Papenbrock *et al.*, 2015). FD_Mine references an *Apriori_Gen* method ( Agrawal *et al.*, 1996) stating that for each pair of candidates *p*, *q* ∈ *C _k_*_–1_ the set *p* ∪ *q* is to be placed in *C _k_* if **card**( *p* ∪ *q*) = *k*. Example 1 shows that the *Apriori_Gen* method referenced and utilized by FD_Mine can violate minimality pruning by checking supersets that need not be checked. Figure 2 gives the power set lattice of the relation described in Example 1 pruned by FD_Mine.

**Example 1.** Let *r*( *U*) be a relation such that *U* = { *A*, *B*, *C*, *D*, *E*}. Suppose that *AB* is a key and that there are no other FDs in *r*( *U*). Since *AB* is a key, we know by definition that *AB* ∪ AB* = *U*. Provided this and that there are no other FDs in *r*( *U*), the candidates *ABC*, *ABD* and *ABE* are deleted from *C*_3_, and so *C*_3_ = *Prune*( *Apriori*_ *Gen*( *C*_2_)) = { *ACE*, *BCE*, *ACD*, *BCD*, *ADE*, *CDE*, *BDE*} ^8^. Then, *C*_4_ = { *ABCD*, *ABCE*, *ACDE*, *ABDE*, *BCDE*}. Because it must be that AB* = { *C*, *D*, *E*}, the algorithm validates the FDs *ABCD* → *E*, *ABCE* → *D*, and *ABDE* → *C*. Since *E*, for example, is functionally dependent on the proper subset *AB* ⊆ *ABCD*, *ABCD* → *E* is non-minimal.

The *Apriori_Gen* principle presented in TANE ( Huhtala *et al.*, 1999) more effectively generates candidate level *C _k_*_+1_ from *C _k_*. It requires that *C _k_*_+1_ only contains the attribute sets of size *k* + 1 which have all their subsets of size *k* in *C _k_* ( Huhtala *et al.*, 1999); i.e.,

$$C_{k+\text{1}}=\left\{X|\text{card}\text{(}X\text{)}=k+1\;\text{and}\;\text{for}\;\text{all}\;ϒ\;\text{with}\;ϒ\;\subseteq \;X\;\text{and}\;\text{card}\;\text{(}ϒ\text{)}=k\;\text{we}\;\text{have}\;ϒ\;\in \;C_{k}\right\}\cdot$$

In reference to Example 1, this method does not insert the candidate *ABCD* in *C*_4_, without loss of generality, because *ABC* ⊆ *ABCD* but *ABC* ∉ *C*_3_. Thus, the non-minimal FD *ABCD* → *E* is not checked.

**Prune**( *C _k_*, *E*, *Closure*) ^9– 11^

$\begin{array}{l} 01\;\text{for each}\;S\in C_{k}\text{:}\\ 02\;\text{for each}\;\;X\in oneDown\;[C_{k}]\text{:}\\ 03\;\text{if}\;(X\;\subset S)\;\text{then:}\\ \text{04}\;\text{if}\;(X\in \{Z\;|\;ϒ↔Z\in E\;\}\;)\;\text{then:}\;\#\;\text{Pruning rule 1}\\ \text{05}\;\text{delete }\;S\;\text{from }\;C_{k}\\ \text{06}\;\text{if}\;S\subset X^{+}\;\text{then:}\;\#\;\text{Pruning rule 2}\\ 07\;\text{delete }\;S\;\text{from }\;C_{k}\\ 08\;S^{+}=S^{+}\cup X^{*}\;\#\;\text{Pruning rule 3}\\ \text{09}\;\text{if}\;U==S^{+}\;\text{then:}\;\#\;\text{Pruning rule 4}\\ \text{10}\;\text{delete }\;S\;\text{from }\;C_{k}\\ 11\;\text{return}\;C_{k},\;Closure; \end{array}$

FD_Mine will under the circumstance described in Example 1 set closure values incorrectly. In line 2, FD_Mine iterates through *C _k_*_–1_, as opposed to *oneDown* [ *C _k_*], which can cause the *Prune*() function to ignore setting the closure values of certain candidates. In Example 1, FD_Mine does not accurately set the closure ABCD* to *E*, since *E* is not saved to the closure values of the candidates *ACD*, *BCD* ⊆ *ABCD* at the previous level. Iterating through *oneDown* [ *C _k_*] sets the closure of a candidate to the union of the closure values of its proper subsets, so that the closure values of deleted candidates are not lost among their supersets.

Properly assigned closure values can allow the algorithm to avoid checking many non-minimal FDs. This is because the *ObtainFDs* module, i.e., the validation method, only checks ^12^ the right-hand side attributes *v _i_* for which *v _i_* ∈ *U* \ *X* ^+^ ( Yao & Hamilton, 2008). Hence, provided that Pruning rule 3 asserts the equality ABCD* = *E*, *ABCD* → *E* need not be checked.

## Operation

FDTool ( Buranosky, 2018) is a command line Python application executed with the following statement: $ fdtool /path/to/file ^13^. For Windows users, this is to be run from the working directory of fdtool.exe, which will likely be C:\Python27\Scripts for those installing with pip install fdtool. For other systems, installation automatically inserts the file path to the fdtool command in the PATH variable. /path/to/file is the absolute or relative path to a .txt, .csv, or .pkl file containing a tabular dataset. If the data file has the extension .txt or .csv, FDTool detects the following separators: comma ( ‘,’), bar ( ‘|’), semicolon ( ‘;’), colon ( ‘:’), and tilde ( ‘∼’). The data is read in as a Pandas data frame _14_.

Dependencies:

1. Python2 ( https://www.python.org/), recommended version 2.7.8 or later.
2. Pandas data analysis library ( https://pandas.pydata.org/) via: pip install pandas.

**Figure 3.**
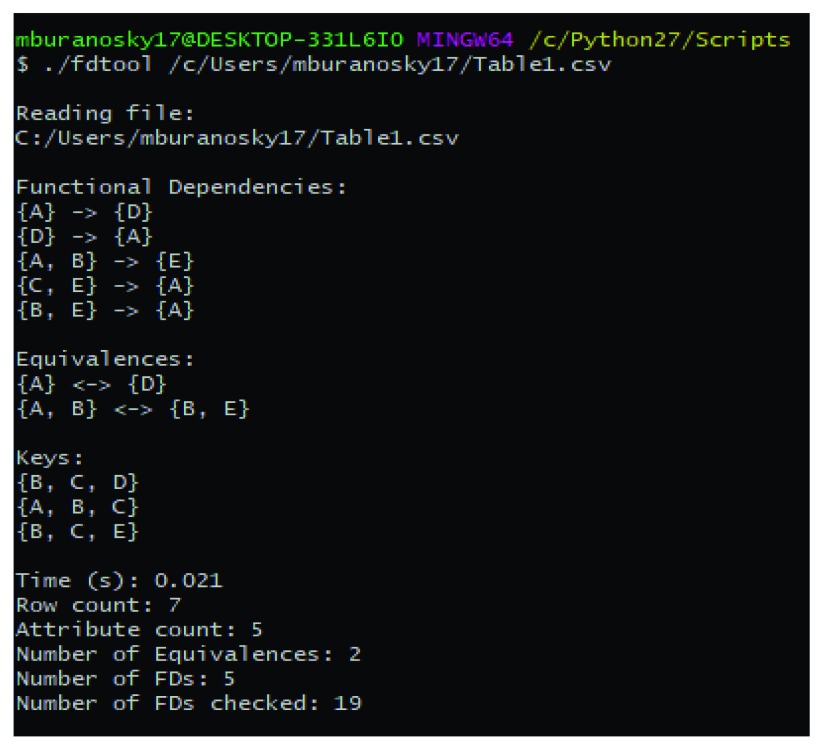
Printed output of FDTool.exe.

FDTool provides the user with the minimal FDs, equivalent attribute sets and candidate keys mined from a dataset. This is given with the time (s) it takes for the code to terminate (after reading in data), the row count and attribute count of the data, the number of FDs and equivalent attribute sets found, and the number of FDs checked. This is printed on the terminal after the code is executed as shown in Figure 3. The information is saved to a .FD_Info.txt file.

Figure 3 shows the printed output of FDTool.exe applied to the contents of Table 1. The output file Table1. FD_Info.txt is saved to the user’s current working directory.

**Table 1.** Example dataset.

A	B	C	D	E
0	0	0	2	0
0	1	0	2	0
0	2	0	2	2
0	3	1	2	0
4	1	1	1	4
4	3	1	1	2
0	0	1	2	0

## Implementation

FDTool is a Python based re-implementation of the FD_Mine algorithm with additional features added to automate typical processes in database architecture. FD_Mine was published in two papers with more detail given to the scientific concepts used in algorithms of its kind ( Yao *et al*., 2002; Yao & Hamilton, 2008). The two versions of FD_Mine were released with different structures but make use of the same theoretical foundation ( Papenbrock *et al.*, 2015), which is fully supported in mathematical proofs of the pruning rules used ( Yao & Hamilton, 2008). FDTool was coded ^15^ with special attention given to the pseudo-code presented in the second version of FD_Mine ( Yao & Hamilton, 2008).

The Python script dbschema.py in FDTool/fdtool/modules/dbschema is taken from *dbschemacmd* ( https://www.elstel.org/database/dbschemacmd.html.en): a tool for database schema normalization working on functional dependencies ( Elmasri & Navathe, 2011). It is used to take sets of FDs and infer candidate keys from them. The operation first assigns the left-hand side attribute combinations of a set of FDs to dictionary keys and their closures to the corresponding values. It then reduces the set of FDs to a minimum coverage ^16^. Candidate keys are assembled using the minimum coverage and closure structure by adding attributes to key candidates until each minimal attribute set X for which *X* ^+^ = *U* is found. Details on the dbschema operations are described in FDTool/fdtool/modules/dbschema/Docs.

## Use cases

FDTool was initially created to help decompose datasets of medical records as part of Clinical Archived Records research for Environmental Studies (CARES). CARES currently contains 13 datasets obtained from the medical software firms Epic and Legacy. The attribute count in this database ranges from 4 to 18; the row count ranges from 42,369 to 8,201,636.

### Experimental results

To limit the strain on computational resources, FDTool has a built in time limit of 4 hours. FDTool reaches this preset limit (triggering program termination) when applied to the PatientDemographics dataset (42,369 rows × 18 columns) and the EpicVitals_TobaccoAlcOnly dataset (896,962 rows × 18 columns). The remaining 11 CARES datasets are given in Table 2 ^17^.

**Table 2.** Experimental results of FDTool on 11 CARES datasets, which terminate in less than 4 hours (preset limit).

Dataset Name	Attribute Count	Row Count	No. of FDs checked	No. of FDs found	No. of Equivalences	Time (s)
AllDxs	7	8,201,686	112	7	1	577.9
AllLabs	10	1,294,106	818	43	4	999.8
AllVisits	9	2,019,117	346	44	7	804.1
EpicMeds	10	1,281,731	453	26	0	551.9
EpicVitals2015	7	1,246,303	127	2	0	86.5
EpicVitals2016	7	988,327	127	2	0	63.0
FamHx	4	93,725	15	0	0	0.7
LegacyIPMeds	8	647,122	79	14	1	28.2
LegacyOPMeds	7	740,616	33	18	1	7.7
LegacyPayors	4	1,465,233	15	4	1	9.4
LegacyVitals	8	1,453,927	146	7	0	134.4

### Experimental summary

The results from Table 2 show that runtime is primarily determined by the number of attributes in a dataset. For instance, the LegacyPayors dataset (1,465,233 rows × 4 columns) has slightly more rows (13% increase) but far fewer attributes (60% decrease) as compared to the AllLabs dataset (1,294,106 rows × 10 columns). The runtime of LegacyPayers (9.4 s.) is much less than that of AllLabs (999.8 s.), because AllLabs has many more arrows in its powerset lattice,

$$n.\;2^{n–1}\;–\;n\;=\;10\;.\;2^{10–1}\;–\;10\;=\;5110,$$

than does LegacyPayers (28). Hence, FDTool has more FDs to check when applied to AllLabs. It is clear that the attribute count of a dataset has a much greater effect on the runtime of FDTool than does row count.

Many of the arrows in the powerset lattice of a candidate are pruned by FDTool. AllLabs has 5,110 arrows in its powerset lattice. However, FDTool only checks 818 FDs, as there are many inferred from the 43 FDs found. This follows from the *Prune()* function, which deletes many of the candidates to check partially as a result of mining 4 equivalent attribute sets. FDTool terminates after 5 *k*-levels when applied to AllLabs.

## Future development

We want to improve its performance so that FDTool is better equipped to handle datasets of different dimensions. Using the dependency induction algorithm FDEP, the reach of FDTool could be extended to datasets with fewer rows and more than 100 columns ( Papenbrock *et al.*, 2015). This might also require upgrading the source code with multicore processing methods, such as a Java API, to reduce runtime and avoid reaching memory limits. A formal proof of the dbschema operations is also desired.

Another goal is to increase the functionality provided by FDTool. This would mean implementing the pen and paper methods typically used to normalize relational schema and decompose tables. Our intent is to incorporate these changes in newer versions of FDTool, released at regular periods, so as to develop it as Python software that could automate much of what is done in the database design process.

## Data availability

Zenodo: USEPA/FDTool: FDTool. https://doi.org/10.5281/zenodo.1442842 ^3^.

While the authors fully support the open dissemination of data for verification and replication purposes, CARES data cannot be released as it contains Protected Health Information. For the purpose of testing the runtime and memory behavior of FDTool, we have produced simulated copies of all 13 datasets in the CARES collection. These datasets are publically available in FDTool/data/input/CARES as part of the FDTool repository and archived in the above Zenodo project. 

Data are available under the terms of the Creative Commons Attribution 4.0 International license (CC-BY 4.0).

## Software availability

**FDTool is available from the Python Package Index:** https://pypi.org/project/fdtool/

**Latest source code:** https://github.com/USEPA/FDTool.git

**Source code at time of publication:** https://doi.org/10.5281/zenodo.1442842 ^3^

**License:** CC0 1.0 Universal. Module FDTool/fdtool/modules/dbschema released under a modified C-FSL license.

## Footnotes

^1^Each attribute *v*_*i*_ has a finite domain, written *dom*( *v*_*i*_), representing the values that *v*_*i*_ can take on. For a subset *X* = { *v*_*i*_,…, *v*_*j*_} of *U*, we write *dom*( *X*) for the Cartesian product of the domains of the individual attributes in *X*, namely, *dom*( *X*) = *dom*( *v*_*i*_) × … × *dom*( *v*_*j*_) ( Yao & Hamilton, 2008). A *relation* r on *U*, denoted *r*( *U*), is a finite set of mappings { *t*_1_,…, *t*_*n*_} from *U* to *dom*( *U*) with the restriction that for each mapping *t* ∈ *r*( *U*), *t*[ *v*_*i*_] must be in *dom*( *v*_*i*_), 1 ≤ *i* ≤ *m*, where *t*[ *v*_*i*_] denotes the value obtained by restricting the mapping *t* to *v*_*i*_. Each mapping *t* is called a *tuple* and *t*( *v*_i_) is called the *v*_*i*_-value of *t* ( Maier, 1983).

^2^FDTool uses Theorem 1 as means to check FDs in the GetFDs module with the *pandas* data analysis library functions *nunique()* and *dropduplicates.count()*.

^3^FDTool saves the closure of candidates at each level before releasing it from memory at levels that follow.

^4^We say that an FD is *checked* when Theorem 1 is used to see if it holds or not ( Yao *et al.*, 2002).

^5^ Definition 5.

^6^ Theorem 1.

^7^An FD X → *A* is *non-trivial* if and only if *X* ∉ *A* ( Huhtala *et al.*, 1999).

^8^Since *E* = ∅ in this example, we can ignore the argument *E* in the function *Prune*(). For simplicity’s sake, we ignore the argument *Closure*.

^9^Closure = { *X* ^+^ | *X* ∈ *C*_k_ V *X* ∈ *oneDown* [ *C*_k_]}.

^11^All candidates at level *k* are stored in *C _k_*.

^12^Assume the left-hand side attribute set *X*.

^13^Edit FDTool/fdtool/config.py prior to building setup with python setup.py install to change preset time limit or max *k*-level.

^15^FDTool was tested regularly throughout the implementation process so as to accomodate to changes made to improve runtime and memory behavior.

^16^A set of FDs F is a *coverage* of another set of FDs *G* if every FD in *G* can be inferred from *F*; i.e., *G* ^+^ ⊆ *F* ^+^ ( Soule, 2014). *F* is a *minimum coverage* of *G* if *F* is the smallest set of FDs that covers *G* ( Soule, 2014).

^17^ **OS**: Windows 10; **Installed memory (RAM)**: 256 GB; **Processor**: Intel Core, 1 CPU; **Clock speed**: 2.19 GHz; **Python**: 2.7.12; **Pandas**: 0.18.1.
