# Supplementary material for: FDTool: a Python application to mine for functional dependencies and candidate keys in tabular data
Source: F1000Res. 2019 Jun 19;7:1667. Originally published 2018 Oct 19. [Version 2] doi: 10.12688/f1000research.16483.2 (PMC6489977; doi:10.12688/f1000research.16483.2)
Supplement: Peer review history

# Peer review: Reviewer response for version 1

**DOI:** 10.5256/f1000research.18017.r46574

**Published:** 2019-04-29

**Authors:** Sayan Mukherjee

**Affiliations:** 1 Department of Statistical Science, Duke University, Durham, NC, USA

This paper outlines in detail FDTool: a Python application to mine for functional dependencies. The paper is about developing a database tool that improves FD Mine a previous tool for functional dependencies

 The definitions, problem statement and explanations in this paper are well written and clear. The authors also provide a good comparison of several functional dependence discovery algorithms. 

 In my opinion, it would be nice to have the experimental results and summary in some more detail. Also since the CARES data is not public it would be nice to have some results on available data.

I confirm that I have read this submission and believe that I have an appropriate level of expertise to confirm that it is of an acceptable scientific standard.

# Peer review: Reviewer response for version 1

**DOI:** 10.5256/f1000research.18017.r39685

**Published:** 2018-12-10

**Authors:** Howard J. Hamilton, Shubhashis Shil

**Affiliations:** 1 Department of Computer Science, University of Regina, Regina, SK, Canada; 2 University of Regina, Regina, SK, Canada

The article is well-structured and well-written. It contains nice presentations of appropriate definitions, theorems, algorithms, examples, and use-cases.

 The article has **clear contributions**:

 In the **theory** part: It enhances the FD_Mine algorithm by improving performance and automating typical processes.

 In the **implementation** part: The authors re-implement the FD_Mine algorithm, which is otherwise not publicly available as a software tool.

 In the **experiment** part: The authors apply FDTool to 12 datasets of different dimensions.

**Findings**: The effect of the attributes is greater than the records on the runtime and memory costs of the FDTool.

          

**Additional contributions**:

 The article clearly describes the features of the FDTool, such as its usage and execution. It also depicts future research opportunities with respect to the further development of the FDTool.

**Major Comment:**

 In the abstract, it says, “We conclude that FD_Mine is the most efficient FD discovery algorithm when applied to datasets with many rows (> 100,000 rows) and few columns (< 14 columns).” The word “conclude” does not seem appropriate here. If this result indeed follows from your research, please explain how the results shown in Table 2 support this claim with respect to all datasets shown in the table [This explanation could be added in the experimental results or experimental summary section]. However, if the conclusion is in fact being taken from Papenbrock, then wording might be adjusted to “Previous research established that FD_Mine ….” You may want to state your conclusions about your software tool.

**Minor corrections:**

 Please use either " **FD_Mine**" or " **FD Mine**" everywhere. The original paper had FD_Mine.

 Please use same format (comma or no comma) for large numbers [e.g. “the row count ranges from **42,369** to **8,201,636**” in the use cases section versus “AllLabs dataset ( **1294106**” and “AllLabs has **5110** arrows in its powerset lattice” in the experimental summary]. Also please check the experimental results section.

 In Definition 2, please correct “ **i 1≠ j**”, presumably to “i ≠ j”.

 In the text immediately after Definition 6, please change “ **determines equivalent attribute sets with**” to “determines attribute sets equivalent to”.

 In the first line of the closure section, please change “ **lead**” to “led”.

 In the future development section, please remove the extra "the" from "A formal proof of **the the** dbschema operations is also desired".

 Table 1 has no title. **Please give a title for Table 1.**

 Table 2 shows 11 datasets but the table title [and the description in the experimental results section] mentions 10 datasets. **Could you please correct this inconsistency.**

We confirm that we have read this submission and believe that we have an appropriate level of expertise to confirm that it is of an acceptable scientific standard.
